# Understanding changes to life expectancy and inequalities in the UK, Germany, and other high-income countries

**DOI:** 10.1007/s00103-025-04126-1

**Published:** 2025-09-09

**Authors:** Gerry McCartney, David Walsh

**Affiliations:** 1 School of Social and Political Sciences, University of Glasgow, Glasgow, Scotland UK; 2 School of Health & Wellbeing, College of Medical, Veterinary and Life Sciences, University of Glasgow, Clarice Pears Building, 90 Byres Road, G12 8TB Glasgow, UK

**Keywords:** Life expectancy, Inequalities, Austerity, Poverty, High-income countries, Lebenserwartung, Ungleichheiten, Sparpolitik, Armut, Länder mit hohem Einkommen

## Abstract

The rate of improvement in life expectancy and mortality slowed considerably in a number of high-income countries from the early 2010s, predating the COVID-19 pandemic by almost a decade. Evidence for different countries, including the separate nations of the United Kingdom (e.g. Scotland and England), shows that this overall ‘stalling’ of improvement has been driven by markedly worsening mortality rates among poorer populations, thereby considerably widening spatial inequalities. Here we synthesise international data and evidence—with a particular focus on the United Kingdom and Germany—to highlight the common causes of these trends, most notably economic ‘austerity’ policies that were implemented in the aftermath of the 2007/2008 financial crash. These have demonstrably increased rates of poverty, reduced availability of required social services, and left public services more threadbare, all of which has negatively impacted mental and physical health and mortality. We conclude with a discussion of the economic policy responses required to address this multi-nation population health emergency.

## Abstract (de)

In Ländern mit hohem Einkommen haben sich der Anstieg der Lebenserwartung und der Rückgang der Sterblichkeit seit Anfang der 2010er-Jahre, also fast ein Jahrzehnt vor der COVID-19-Pandemie, teilweise erheblich verlangsamt. Daten aus den betroffenen Ländern, darunter auch aus den einzelnen Teilen des Vereinigten Königreichs (z. B. Schottland und England), verdeutlichen, dass die Stagnation der Lebenserwartung auf eine deutliche Verschlechterung der Sterblichkeitsraten in ärmeren Bevölkerungsgruppen zurückzuführen ist, wodurch sich die räumlichen Ungleichheiten erheblich vergrößert haben. Hier fassen wir internationale Daten und Erkenntnisse zusammen – mit besonderem Schwerpunkt auf dem Vereinigten Königreich und Deutschland –, um die gemeinsamen Ursachen dieser Trends aufzuzeigen, insbesondere die wirtschaftlichen „Sparmaßnahmen“, die nach der Finanzkrise von 2007/08 umgesetzt wurden. Diese haben nachweislich die Armutsquote erhöht, die Verfügbarkeit notwendiger sozialer Dienstleistungen verringert und die öffentlichen Dienstleistungen ausgehöhlt, was sich negativ auf die psychische und physische Gesundheit sowie die Sterblichkeit ausgewirkt hat. Wir schließen mit einer Diskussion über die wirtschaftspolitischen Maßnahmen, die erforderlich sind, um diese multinationale Gesundheitskrise zu bewältigen.

## Introduction

Since around 2012, the previously observed steady improvements in life expectancy and mortality rates across high-income countries have stalled, such that there has been slower improvement in countries such as the Netherlands and almost no improvement in others, including Australia, Germany, the United Kingdom (UK), and the United States [1–3].

There has been substantial debate about the causes of these changed trends (including a focus on specific diseases such as influenza, dementia, and cardiovascular disease) and the priority that should be given to them [2–5]. However, it is now clear from the accumulation of a substantial body of evidence that economic policies implemented across countries have been the key underlying cause of this change in trends [2, 3, 6]. This article synthesises the available evidence in order that the scale, urgency, causes, and appropriate responses are clear to policymakers and researchers. We do so with a particular focus on the UK (including Scotland, where the authors are based) and Germany (given the likely interests of the journal’s readers).

## Epidemiology of the stalled trends

Life expectancy (alongside the related measure of age-standardised mortality rates) is a summary measure of population health that reflects mortality risk across all age groups and allows comparison over time and between places. Life expectancy had steadily improved for men and women for most high-income countries since 1950 [2, 7]. However, around 2012 these trends changed across many (but not all) high-income countries, with many experiencing a much slower rate of improvement or even a decline in some years [1, 2, 8]. As Table 1 (which sets out the key phases and characteristics of the trends in many high-income countries) shows, the changes predate the COVID-19 pandemic but were made worse by it. The deficit in life expectancy compared to what would have been expected is now substantial, due to a steady deviation from previous trends rather than the influence of any specific year [9].

**Table 1:** Generalised characterisation of the nature and causes of the changing mortality trends in high-income countries

Phase 1 (2000–2012)	Generally improving with mixed inequality trends [10]
Phase 2 (2012–2020)	Widespread stalling of trends with widening inequalities [7, 8, 11]
Phase 3 (2020–2021)	Worsening trends with widening inequalities associated with the acute COVID-19 period [12]
Phase 4 (2022 onwards)	Return to prepandemic stalled trends and widening inequalities associated with ongoing austerity and additional high inflation [7, 13]

**Fig. 1:**
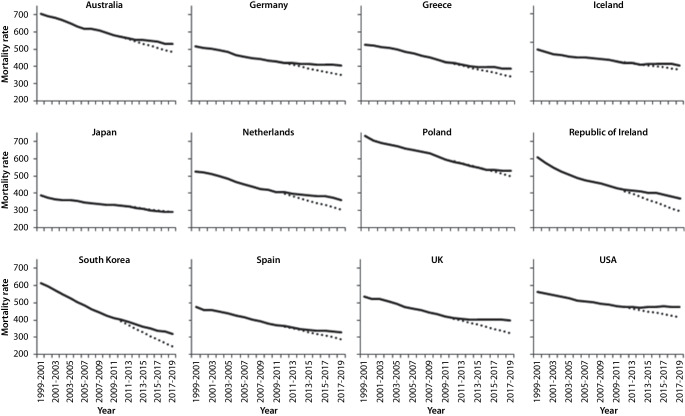
Age-standardised mortality rates for selected high-income countries, 1999–2001 to 2017–2019, showing both observed (solid line) and projected (dotted line) rates. Source: Walsh & McCartney, 2025 [2]

To illustrate this point, Fig. 1 shows trends in age-standardised mortality rates for a selection of high-income countries. The dotted line represents the rates that would have been anticipated had previous trends continued. A divergence between projected and observed rates can be seen in the majority of the 12 countries shown. However, the extent of the divergence varies, and not all countries are affected: The trend in Japan—where mortality rates were already lower than in the other countries prior to the changes taking place—continued on the previous trajectory [2].

**Fig. 2:**
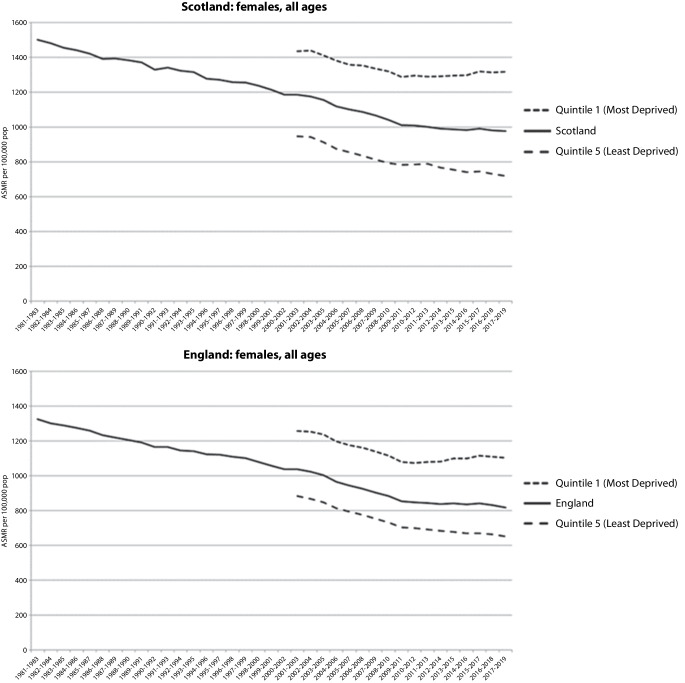
Age-standardised mortality rates (ASMRs) for females of all ages in Scotland and England, including their 20% most and least deprived populations, 1981–2019. Source: Walsh & McCartney, 2025 [7]

The trends in the UK and Germany are very similar. In the UK, detailed analyses of the composition of the changes have been undertaken. Across all four UK nations (Scotland, England, Wales, and Northern Ireland), deteriorations in mortality improvement have been observed for men and women, all age groups, and almost all specific causes of death, with particularly large contributions due to slower improvements in cardiovascular mortality and increased mortality from drug-related deaths and dementias [14, 15]. Some initial commentaries pointed towards particularly substantive changes amongst older age groups, but this was either based on year-to-year changes rather than analysis of the trends or did not consider the greater impact on overall life expectancy by people dying at younger ages [2, 3]. Importantly, the overall stalling in mortality trends masked a deterioration amongst people living in the most deprived circumstances in each of the UK nations [7, 8]. Figure 2 shows a clear widening of spatial inequalities in both Scotland and England; these data are for females, but trends are similar for males. There is also some evidence that overall morbidity and mental health may have worsened [16, 17].

## Economic policy as the key cause of the changed trends

Despite substantial initial speculation about the causes of the changed trends across countries [14], it is now clear that economic policy changes, and specifically the introduction of austerity measures [2, 18], have been the key underlying cause [2, 3], working through well-understood causal pathways [18]. Austerity is ‘characterised by reductions in government spending and/or increases in taxation after accounting for “automatic stabilisers” in the economy (such as changes in tax revenues or payment of unemployment benefits, which vary depending on whether there is economic growth or recession)’ [18]. Many countries implemented forms of austerity in the decade following the 2007–2008 financial crash, including the UK and Germany, but with differing forms, timings, and extents [2, 6].

In the UK, austerity was implemented incrementally from 2010 following a change of government. It involved substantial and sustained reductions in public spending, particularly on local government and social security [2, 3, 19, 20]. This led to a marked real-terms reduction in the value of welfare benefits, increased conditionality, and increased sanctions, leading many people to have reduced or zero incomes for prolonged periods of time. The detrimental mental health and mortality consequences of some of these changes have been well documented [21, 22]. The negative impact of local authority budget reductions in England on mortality has been large [23]. Similar consequences have been observed from cuts to social security payments and entitlements across Scotland, England, and Wales [24]. At this time there was also continued broader restructuring of the economy, which led to an increase in employment precarity and in-work poverty, further contributing to the damaging economic exposures [18, 25]. Austerity policies in the UK have been shown to have impacted a whole range of health determinants and outcomes: increased child poverty (greater than in any Organisation for Economic Co-operation and Development country [26]), greater ‘food insecurity’ (hunger, foodbank dependence), higher levels of homelessness, worsening mental health, decreased *healthy* life expectancy, and more births of premature and low birthweight babies, as well as the changes to mortality rates and life expectancy already discussed [2].

While some commentators have argued that Germany has not implemented austerity policies, and thus its slowdown in mortality improvements has been used as an argument against austerity being the cause of changed trends in other countries, [2], this is not supported by the evidence. The data show clearly that following the financial crash of the late 2000s, economic policy was shaped by austerity measures between 2012 and 2017 [2, 6]. Furthermore, during a temporary increase in public spending at the onset of the COVID-19 pandemic in early 2020, it was made clear by the German government itself that it would be returning to its favoured ‘austere’ approach as soon as possible: ‘Once the crisis is over—and we hope this will be the case in several months—we will return to austerity policy’ [27]. As was the case in the UK, the nature of that policy included changes to eligibility for social security payments and harsher financial ‘sanctions’ for those falling foul of the system, both of which disproportionately affected immigrants [28]. Also, as in the UK, local government funding was cut, affecting the availability and cost of public services [29]. Furthermore, like the UK and other countries such as the United States, these actions followed on the heels of a much longer-term ‘neoliberal’ policy approach involving privatisation, welfare state reduction, decreased worker protection, and declines in the real value of social security benefits; all these measures are associated with widening socioeconomic and health inequalities [2]. Poverty, and in particular extreme poverty, and income inequalities in Germany have now reached record levels [30, 31].

In Germany, both long-term and shorter-term policy approaches had consequences for health, such as stalling improvements in life expectancy overall and widening the health inequalities measured at individual and spatial levels [6, 32, 33]. The role of economic policy in these changes has not been at the forefront of research and policy analysis; rather, the focus has erroneously been on health care, demographics, and individual behaviours [2, 34–36].

## The contribution of specific causes of death and risks

As noted previously, the changed trends in mortality have been observed across countries [1–3], age groups, sexes, and almost all causes of death [14, 15]. Economic exposures can explain these widespread impacts, whereas explanations that relate only to specific causes of death or disease processes are much more limited [2, 3, 37]. However, the rise in obesity during the 1990s and 2000s across many high-income countries, but particularly the UK and the United States, is likely now to be making a contribution to the stalled trends. Obesity carries higher risks of a wide range of conditions, including cardiovascular disease and cancer, and it has been estimated that it might explain a proportion of the stalled trends due to lagged impacts (from 10% of the stalling for men in Scotland to 35% of the stalling for women in England, although these figures are uncertain and may be overestimates) [38]. However, as with all attributable fraction estimates, other exposures are also likely to be important. The rise in obesity prevalence up to around 2010 was due to the creation of an obesogenic environment through, for example, changes in food marketing and availability and changes in urban and working environments.

**Fig. 3:**
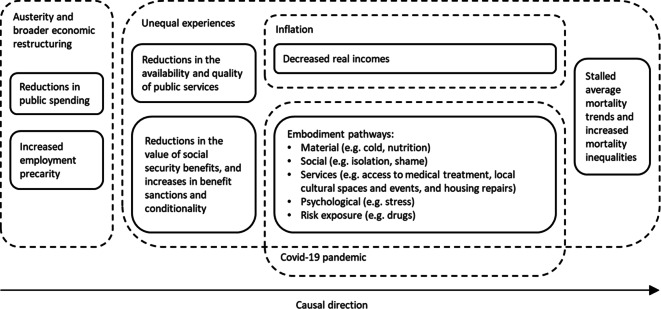
Schematic outlining the mechanisms linking austerity to stalled mortality trends

On the other hand, suggestions that specific causes of death such as influenza [39], ‘deaths of despair’ (drug, alcohol, and suicide deaths) [40], or cardiovascular disease are largely responsible for the changed trends carry risks of misdirection [2–4]. Any condition-specific explanation can only be partial given that the stalling in all-cause mortality is due to changes across almost all causes of death and across all age groups. For these to be plausible and sufficient explanations, independent causal processes for all of these conditions must have occurred at the same time and across countries. Instead, consideration of how economic changes have simultaneously had an impact on a wide range of disease-specific processes, embodied through a range of material and psychological processes [41], is a substantially more credible approach (Fig. 3). Furthermore, the contribution of some of these specific causes of death (particularly influenza and drug-related deaths) to the overall population changed trends is small [14, 15]. Mortality linked to influenza has been declining for many years and has continued to do so [14, 37]. Drug-related deaths do contribute more substantially to the changed trends in younger adults, particularly in Scotland and the United States [38, 42]. However, drug-related deaths are also very sensitive to changes in the economic context and represent an important mechanism linking austerity and all-cause mortality [43, 44]. Regarding cardiovascular disease, there has been little argument made as to why previously improving trends have stalled, with calls for better data or renewed focus on health behaviours rather than any contextualised understanding of how cardiovascular disease trends are influenced by changes in the economic context [3, 45].

## COVID-19 and the costs-of-living crisis

As stated, the changed trends in mortality predate the COVID-19 pandemic but have been exacerbated as a result. It is likely that both the direct impacts of the infection, as well as the indirect consequences of the infection control measures (including the social, economic, and health care disruption), have affected health and mortality risk [13]. As countries have emerged from the initial pandemic waves and economies have opened back up, high inflation has led to a decrease in real incomes, especially for people with lower incomes, and this is modelled to have large negative mortality and health consequences and to widen health inequalities [46].

Figure 3 shows how austerity and broader economic restructuring is likely to have impacted some groups disproportionately (e.g. people with disabilities, the working class) through deteriorating public services, decreased value of and eligibility for social security benefits, and decreased real incomes, through a variety of pathways of embodiment [41] and exacerbated by the COVID-19 pandemic, to lead to a prolonged period of stalled mortality across high-income countries. These average trends, however, mask a rapid worsening in mortality trends for people living in the most deprived areas (as shown for Scotland and England in Fig. 2).

## Conclusions and implications for policy and research

Even before the COVID-19 pandemic, the rate of improvement in mortality across many high-income countries slowed substantially [2, 3, 6, 14]. Changed trends in almost all causes of death and across all age groups contributed [14, 15, 47]. The resulting loss of life was substantially greater than that due to the pandemic [48] but has gained much less public attention and policy action. It is now clear that economic policy changes, in particular austerity measures, have been the most important cause of these changed trends [3, 6]. However, this general economic policy approach remains dominant in many countries, compounding the challenges of the costs of living and the ongoing impacts from the pandemic [46, 49].

Governments should reassess economic policy design, increase funding for public services, increase the value of social security benefits, and reduce the precarity and conditionality of incomes from benefits and work in order to support improvements in population health. This would make population health a key objective of economic policy, in contrast to the current approaches that see economic growth as the long-term objective, and improved population health as a means to that end [50].

## Declarations

For this article no studies with human participants or animals were performed by any of the authors. All studies mentioned were in accordance with the ethical standards indicated in each case.
